# Precarious employment and migrant workers’ mental health: a systematic review of quantitative and qualitative studies

**DOI:** 10.5271/sjweh.4019

**Published:** 2022-06-30

**Authors:** Ozlem Koseoglu Ornek, Julia Waibel, Pia Wullinger, Tobias Weinmann

**Affiliations:** 1 Institute and Clinic for Occupational, Social and Environmental Medicine, University Hospital, LMU Munich, Munich, Germany

**Keywords:** anxiety, depression, discrimination, job insecurity, temporary employment, transient, occupational health, temporary worker, stress

## Abstract

**Objectives:**

Evidence suggests that precarious employment can have detrimental effects on workers’ health, including mental health. Migrant workers are discussed to be especially vulnerable to such effects. Thus, we systematically reviewed existing research on the association between precarious employment and migrant workers’ mental health.

**Methods:**

Three electronic databases (Web of Science, PsycINFO and PubMed/Medline) were searched for original articles on quantitative and qualitative studies published from January 1970 to February 2022 in English, German, Turkish and Spanish. Multiple dimensions of precarious employment were considered as exposure, with mental health problems as outcomes. Narrative synthesis and thematic analyses were performed to summarize the findings of the included studies along with risk of bias and quality assessment.

**Results:**

The literature search resulted in 1557 original articles, 66 of which met the inclusion criteria – 43 were of high quality and 22 were of moderate quality. The most common exposure dimensions analyzed in the studies included temporariness, vulnerability, poor interpersonal relationships, disempowerment, lacking workers’ rights and low income. The outcome measures included stress, depression, anxiety and poor general mental health. The prevalence of these outcomes varied between 10–75% among the included quantitative studies. All qualitative studies reported one or more dimensions of precarious employment as an underlying factor of the development of mental health problems among migrants. Of 33 quantitative studies, 23 reported evidence for an association between dimensions of precarious employment and mental health.

**Conclusion:**

The results of this review support the hypothesis that precarious employment is associated with migrant workers’ mental health.

Globally, the number of migrants is increasing steadily due to climatic disasters, globalization, socio-political pressures and conflicts (1, 2). According to the International Labor Organization (ILO), there are approximately 258 million international migrants in the world, 164 million of whom are workers. About two-thirds of these workers live in high-income countries where they are an important labor resource (3). However, migrant workers tend to contribute to the labor force in their host countries through precarious employment, which is characterized by unfavorable work conditions. These conditions include job insecurity, low income, lack of worker rights and protection, lack of power to exercise rights, work with temporary or no contracts, participation in multiple part-time jobs, lack of employment compensation, unpredictable working schedules, and exposure to unfair and authoritarian treatment (4–10).

Precarious employment has become more prominent recently, especially in high-income countries (4, 9). Moreover, the impact of the COVID-19 pandemic has the potential to further increase the prevalence of precarious working conditions (11, 12). Apart from that, there is evidence that precarious employment can have negative effects on workers’ quality of life and well-being (6), ultimately leading to health problems (13–16). For example, some studies indicated an association between precarious employment and physical health problems such as musculoskeletal issues (17), cardiovascular diseases (18), occupational accidents and injury (19, 20). Results of other studies also show that precarious work conditions have an influence on mental health problems such as depression (21), anxiety and stress (22), suicidal ideation (23, 24), sleep issues and burnout (24–26). Accordingly, there are a number of systematic reviews that have synthesized the evidence for detrimental mental health effects of different dimensions of precarious employment (5, 9, 27–31).

However, none of the reviews so far specifically focused on migrant workers. Migrants are especially vulnerable and likely to be exposed to precarious employment because of language barriers, employer prejudice and discrimination, lack of professional networks, lack of sufficient knowledge related to health and the labor system (32–34) and poor social support (35, 36). In addition, migrant workers have been reported to be exposed to various forms of harassment at the workplace: prejudices by employers and workmates (33), unfair treatment and discrimination (37–39) or being forced to work or perform tasks that are incompatible with their contracts at the threat of deportation (37, 40–42). This situation is often worse in the case of undocumented migrants who are even more vulnerable and disadvantaged due to a lack of work or residency permits. As a consequence, they are at risk from being exploited by their employers (38, 40, 41, 43, 44). Moreover, there is considerable evidence that migrants differ from non-migrants in characteristics such as general health status, access to healthcare or health-related risk factors (45, 46).

Because of those peculiarities, studies examining precarious employment and its putative health effects in the general population cannot be automatically and directly transferred to migrant populations. It is thus crucial to comprehensively understand and systematically evaluate migrant workers’ experiences under precarious work conditions and such conditions’ effect on migrant workers’ mental health. Apart from a better scientific understanding, such findings may also help to develop migrant worker-friendly occupational health policies in the future. Thus, the main aim of this review of qualitative and quantitative studies was to analyze and summarize existing quantitative and qualitative research on the association between precarious employment and migrant workers’ mental health. To achieve this aim, the scientific questions were addressed in *quantitative studies:* (i) What is the prevalence of precarious employment among migrants? (ii) What is the association between precarious employment and mental health among migrants, including its direction and effect size? and in *qualitative studies:* (i) What dimensions of precarious employment are migrant workers exposed to? (ii) What are the mechanisms underlying the relationship between precarious employment and mental health among migrant workers?

## Methods

### Protocol

We described our methods by means of a detailed protocol (47) that was developed in accordance with the Preferred Reporting Items for Systematic Review and Meta-analysis Protocols (PRISMA-P) 2015 guidelines (48), and was registered within the International Prospective Register of Systematic Reviews (PROSPERO) (registration number: CRD42019132560).

### Eligibility criteria

The Population, Exposure, Comparator and Outcomes (PECO) framework was used as a guide for the eligibility criteria of the included publications (49). The included papers were original quantitative observational studies and all types of qualitative studies that involved international migrant workers of working age (≥15 years), and published in English, German, Spanish or Turkish in peer-reviewed journals from 1 January 1970 to 14 February 2022. All original studies with ‘precarious employment’ as an exposure and mental health problems as an outcome were included.

### Search strategy and information sources

Keywords were determined on the basis of multi-dimensional precarious employment definitions, eg, the dimensions of precarious employment identified with the Employment Precariousness Scale (EPRES) (8, 50, 51) and an earlier reviewer with a similar scope (5) and related to three main headings, namely, ‘migrant’ (population), ‘precarious employment’ (exposure) and ‘mental health’ (outcome). Search strategies were developed with these keywords using the Medical Subject Headings (MeSH) thesaurus and freetext based on the eligibility criteria (supplementary material, www.sjweh.fi/article/4019, table S1). With these keywords, information sources were searched in the following order. First, three electronic databases (Web of Science, PsycINFO and PubMed/Medline) were searched for original observational studies published from January 1970 to February 2022. Then, we hand-searched the reference lists of previously published related systematic reviews. We also hand-searched for relevant studies published in the *American Journal of Industrial Medicine*, *BMC Public Health, Ethnicity & Health*, and *Gaceta Sanitaria* in the last year from which we obtained the largest number of eligible studies in the database search. We included all 2019 issues that were published online in the hand-search. Lastly, the reference lists of all included studies were screened based on the eligibility criteria.

### Data collection, selection process and extraction

**Figure 1:**
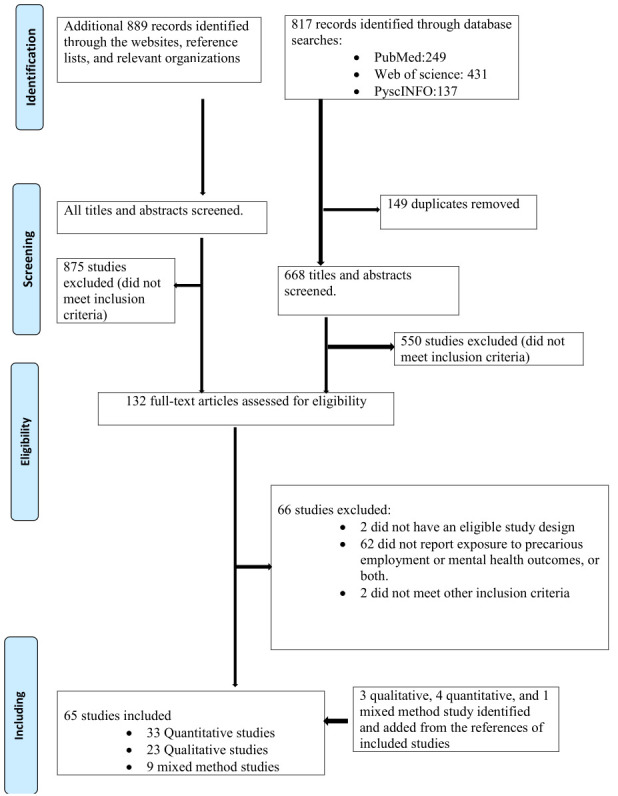
Flowchart of included studies.

First, two authors independently evaluated the titles, and abstracts of the identified articles. Secondly, two authors independently assessed the fulltext of all candidate articles. Any disagreements were reconciled by discussion with consultation of a third researcher to build consensus if necessary. A flowchart showing details about the selection process is illustrated in figure 1. Two reviewers independently used a standardized form for data extraction from each included study in the data collection stage. A third reviewer was consulted for resolution in case of differences or disagreement between the reviewers’ evaluation. We contacted 12 corresponding authors to obtain certain data from their respective studies such as participants’ age or gender distribution if the information was not included in the published manuscript.

### Risk of bias and quality assessment

Two reviewers independently assessed the quality of the included qualitative and quantitative studies. The Newcastle-Ottawa Scale (NOS) was used to evaluate the quality of the quantitative studies (31). The Critical Appraisal Skills Programme (CASP) was used to assess the qualitative studies. The assessed quality of the included studies was graded according to the total score obtained from NOS and CASP. Discrepancies between the reviewers were discussed until a consensus was achieved.

### Data analysis and synthesis

The results of the included studies are reported as a systematic review narrative synthesis. No meta-analysis was conducted due to variations in the included studies’ precarious employment dimensions, mental outcomes and measurement methods. Additionally, for studies that did not report confidence intervals (CI), we calculated the 95% CI using RevMan (version 5.4) (52). Thematic analysis was performed to synthesize the qualitative studies (53) using MAXQDA (54). One researcher developed an initial coding frame that a second researcher then checked. Themes and subthemes emerged on the basis of the initial coding. The results of the qualitative and quantitative studies were decided to be combined according to their common features and contents after discussion and analysis among the researchers. According to this decision, a form was developed based on themes and categories. The results of the review presented following the guidelines for systematic reviews and meta-analyses of observational studies (MOOSE) (55).

## Results

### Study selection

The database search produced 817 results, and the hand search of the websites and reference lists added 899 studies; in total, 1706 studies were acquired (figure 1). After screening for duplicates, 149 papers were removed; the titles and abstracts of the 1557 remaining studies were then assessed. Of these, 668 studies from the database search and 875 studies from the hand search sources were excluded because they did not fulfil the inclusion criteria, leaving 132 studies for full-text reading. Of these, 57 were found to be eligible for inclusion. The reference lists of the included studies were screened and 8 of these papers met the eligibility criteria. Finally, 65 studies were included, of which 33 were quantitative (21–23, 25, 33, 40, 56–82), 23 were qualitative (24, 32, 34, 37, 41, 43, 44, 83–99) and 9 were mixed-methods studies (26, 35, 38, 39, 42, 84, 100–102). However, these studies were found to comply with the inclusion criteria with either only qualitative (35, 38, 39, 42, 84, 101, 102) or only quantitative findings (26, 100).

In total, 65 studies were assessed for quality. About two-thirds of them (26 qualitative, 17 quantitative) were of high quality, while about one-third (4 qualitative, 15 quantitative) were of moderate quality (supplementary tables S2 and S3).

### Study characteristics

Three studies (two quantitative, one qualitative) were published in Spanish, the others in English. None of the studies were published in German or Turkish. The vast majority of studies were conducted in North America (43%) or Europe (38%). The youngest participant age in all the studies was 16, and the majority of participants were female workers.

Nineteen of the quantitative studies consisted of a study population that included migrant and non-migrant workers. Two studies involved the same participants, but provided data on different variables (40, 79). Most of them were cross-sectional studies. They were published between 1998 and 2022, with more than 70% being published after 2009. All but one quantitative studies collected their data after the 2000s. The General Health Questionnaire-12 (GHQ-12) was commonly used to evaluate mental health. Aside from the Employment Precariousness Scale, which was used in three studies (77, 78, 80), there was no specific tool for assessing precarious work conditions (table 1).

**Table 1:** Characteristics of the included quantitative studies. [PR=Prevalence Rate; rpb=Point-biserial Correlation; X^2^=Chi Square; EPRES=the Employment Precariousness Scale; COPSOQ-ISTA21=Spanish Version of Copenhagen Psychosocial Questionnaire; ENETS=Quality of Life and Employment, Labor, and Health Conditions First National Survey; SF-36=Scales from the Spanish Version of the short form Health Questionnaire; GJSQ=Generic Job Stress Questionnaire; RSES: Rosenberg’s Self-Esteem Ecale; CCS=Cybernetic Coping Scale; CES-D 20=Epidemiological Studies Depression Rating Scale; MHI-5=Mental Health Inventory 5; MFWSI=Migrant Farmworker Stress Inventory; PAI=Personality Assessment Inventory; DASS-42=Depression, Anxiety and Stress Scale;JCQ=Job Content Questionnaire; GHQ=General Health Questionnaire; PHQ-9=Patient Health Questionnaire 9; ERI=Effort-reward Imbalance; 4M represents the Spanish version of CAGE, which an instrument for alcoholism screening; WHO= World Health Organization; NA=Not available]

Authors, year, country (reference), Data years	Study population (nationality/ work types)	Age (years)	Gender (F/M)	Study Design	Measures	Statistical Analysis	Exposure	Outcome	Main Results
Agudelo-Suarez et al, 2009 (40), Spain 2008	2434 migrant workers from Colombia, Ecuador, Morocco and Romania working in agriculture, industry, construction, and services	≥18	1039 / 1395	Cross- sectional	GHQ-12, questionnaire	Descriptive analysis	Undocumented status Temporary or no contract Low income Multiple jobs Lack of work rights	Poor mental health	27% of the participants reported mental health problems. 22% had no documents, almost 72% had a temporary contract or no contract, 73% reported low income, 23% were not able to take a medical leave, 22% could not use a weekly rest day, and 32% could not take a leave when they needed, while 23% had no social security.
Agudelo-Suárez et al, 2011(79), Spain 2008	2434 migrant workers from Colombia, Ecuador, Morocco and Romania working in agriculture, industry, construction, and services	≥18	1047/ 1387	Cross- sectional	GHQ-12, questionnaire	Logistic regression	Discrimination at workplace	Poor mental health Stress Anxiety Insomnia	Workers reporting workplace related discrimination were more likely to report poor mental health (aOR 2.97; 95% CI 2.45-3.60), stress (aOR 1.50; 95%CI 1.26-1.79), insomnia (aOR 2.06; 95% CI 1.64-2.60) and anxiety (aOR 1.79; 95% CI 1.44-2.23)
Al-Maskari et al, 2011(23), United Arab Emirates 2008	239 migrant workers from various occupations and different countries	>18	All male	Cross- sectional	DASS-42, questionnaire	Univariate logistic regression	Low income Lack of annual leave	Depression Suicidal ideation	The prevalence of depression among workers was over 25%, low income was over 64%; over 6% indicated suicidal thoughts, 2.5% suicide attempts. Migrant workers with low income were more likely report depression (aOR: 1.80; 95% CI 1.33–3.16) and suicidal ideation (aOR:5.98; 95% CI 1.26-28.45)
Benach et al, 2015(80), Spain 2010	970 employed workers in Catalonia (Spain), 223 of them migrants	≥16	112/ 111	Cross-sectional	EPRES, GHQ-12, questionnaire	Log-binomial regression models	Precarious employment	Poor mental health	Association between precariousness and mental health (in total sample): PR: 3.21 (95% CI 2.08-4.95) for 4th vs. 1st quartile of precariousness. Prevalence of precariousness among migrants: 67.0 (61.6-72.0).
Bhui et al, 2005(81), United Kingdom NA	2054 individuals from different countries including non-migrant workers	Mean: 33±1.0	921/ 1133	Cross-sectional	Revised Clinical Interview Schedule scale	Univariate logistic regression	Discrimination at work (job denial, unfair treatment, insult)	Mental disorders	Job denial (OR:1.8; 95% CI1.2-2.7), unfair treatment (OR:2.5; 95% CI1.6-3.8), and insult (OR:2.3; 95% CI1.4-3.6) were strong predictors for mental disorders.
Burgel et al, 2019(21), United States 2010-2011	130 migrant taxi drivers from 32 different countries	25-71	8/122	Cross- sectional	CES-D ERI, Personal Stressful Life Events Scale, JCQ	X2, Pearson correlation/Spearman Rho Logistic regression	Discrimination/unfairness at work Lack of health insurance	Perceived mental exertion Depressive symptoms	Migrant workers who had no insurance were more likely report depression (OR:4.51; 95% CI 1.28-15.98), and perceived mental exertion (OR:4.52; 95% CI 1.28-15.98). No significant association between discrimination and perceived mental exertion or depression symptoms. 38.5% of the migrants reported perceived mental exertion, and 38% indicated depression.
Cayuela et al, 2015(82), Spain July 2011-June 2012	7880 non-migrant and 710 migrant workers (mostly manual workers)	≥18	356/354	Cross sectional	GHQ-12, questionnaire	Multivariate logistic regression	Working arrangements such as fixed term, temporary contract, no-contract or verbal contract	Poor mental health	Migrant women reported higher probability to experience poor mental health compared to non-migrant women workers (aOR: 2.12; 95% CI1.44-3.12). More migrant men (30%) had a temporary contract than migrant women. The percentage of migrant women (13%) who worked with no contract or verbal contract was more than the number of migrant men (3%), while migrant women reported two times more poor mental health compared to the migrant men. 16% of the migrant men and 30% of the migrant women reported poor mental health.
Chang et al, 2020(56), Taiwan NA	85 home-based migrant care workers	31.4 ± 6.4	85/0	Cross-sectional	Caregiver Strain Index, questionnaire	Univariate and multivariate logistic regression	Inadequate salary	Stress	37.6% of the participants indicated high stress levels. Inadequate salary was reported as a risk factor for psychological stress (OR: 10.14; 95% CI 2.80-36.70)
Daly et al, 2019(57), Australia 2013,2017-18	2215 participants from different countries including non-migrant workers	18-65	892/ 1323	Cross-sectional	Mental Health Inventory Kessler 6	Univariate logistic regression	Jobs with low security Unfair payment Precarious work contracts	Mental health problems	Jobs with low security (aOR: 3.4; 95% CI 2.6-4.4) was a strong predictor for mental health problems. 63% of migrant workers had low job security, and 54% of them reported unfair payment.
Del Amo et al, 2011(58), Spain (60) 2006-2007	554 Spanish-born workers, 568 migrants from Ecuador	18-55	285/ 283	Cross-sectional	GHQ-28, questionnaire	Logistic regression	Short term, temporary, no contract Low wage	Psychiatric case	Possible psychiatric case prevalence was higher in Ecuadorian women (34%; 95% CI 29–40%) compared to non-migrants (non-migrant (24%; 95% CI 19–29%) and Ecuadorian men (14%; 95% CI 10-18%). 8% migrant workers reported to have a low wage, and 15% expressed to have high economic difficulties. The percentage of Ecuadorian who had temporary or no contract was higher compared to non-migrants.
Dhungana et al, 2019 (59), India & Nepal 2017-2018	751 Nepali cross-border migrants working in India	32±9.2	25/726	Cross-sectional	GHQ-12, questionnaire	Poisson regression	No sick leave, No day off Low income	Poor mental health	35.9% of the participants had no sick leave provision, while the prevalence of psychological morbidity was 13.5%. Evidence for an association between no sick leave and psychological morbidity (PR: 2.4; 95% CI 1.32–4.34); no association between provision of days off and mental health (p=0.39)
Drydakis, 2022(60), Greece 2018-2019	Migrant workers from Asia, Africa and Europe (panel 1: N=152; panel 2: N=156; panel 3: N=308)	32.1±7.7 (panel 1) 32.3±7.8 (panel 2) 32.2.1±7.7 (panel 3)	34/118 (panel 1) 42/114 (panel 2) 76/232	Panel study	CESD-20, questionnaire	Random effects models	No contract Low wage Insults/threats in the workplace	Depression	Prevalence of depression ranged from 13.7% (panel 2) to 14.9% (panel 1). More than half of the workers had not written contract. No contract (coef=4.312, p<0.01), wage lower than the national minimum (coef=5.005, p<0.01), and experiencing insults and/or threats at work (coef=3.915, p<0.01) were statistically significantly associated with depression.
Espinoza-Castro et al, 2021(61), Germany 2018-2020	99 aupairs from Latin America and Spain	19-28	87/12	Cohort (6 months of follow-up)	PHQ-9, ENETS, European Working Conditions Survey	Generalized estimating equation (GEE)	Working > 40 hrs. per week Physical violence and verbal offenses at work	Depression	Association between working more than forty hours per week (OR: 3.47; 95% CI 1.46–8.28) and exposure to physical violence from host children (OR: 4.95; 95% CI 2.16–11.34) and symptoms of depression.
Font et al, 2012 (33), Spain October 2004 and July 2005	6868 non- migrants and 687 migrant manual and non-manual workers	16-65	NA	Cross- sectional	COPSOQ. ISTA21, SF-36-Spanish version	Log-binomial model	Job insecurity	Poor mental health	Migrant workers had worse mental health than non-migrant workers (PR: 1.09; 95% CI 1.02-1.16). Prevalence of perceived poor mental health among migrant workers was almost 60%.
Grzywacz et al, 2010(62), United States 2007	288 Latino farm workers, mostly from Mexico	NA	25/263	Cohort (4 months follow- up)	CES-D- 10-item version, MFWSI	Mixed-effect model	Discrimination and marginalization Undocumented status	Depression	24% of participants classified as potential clinical case of depression during the agricultural season. The higher they had concern about undocumented status, discrimination and marginalization the higher symptoms of depression they reported.
Hammond et al, 2010(63), United States NA	664 employees (290 migrants)	≥22	203/87	Case-control	CES-D 20-item version	Chi-square test, Linear regression models	Discrimination at workplace	Depression	Almost 16% of Asian Pacific Islanders and over 11% of the Latino workers reported discrimination at the workplace. Discrimination at workplace was correlated with depressive symptoms among Asian Pacific Islanders (β (SE):-02 (.83),and Latino workers (β(SE):03(1.05).
Haro et al, 2020(64), United States 2004	2015 migrant day labourers	34.2±10.9	NA	Cross-sectional	PHQ-2, questionnaire	Multivariate logistic regression	Underpay- ment/no payment, no breaks (employer abuse)Insult/harassment/threats from business owner (business abuse)	Depression	10% of the participants had a PHQ-2 score that screened for depression; 78.5% reported at least one form of employer abuse (e.g., underpayment). Both employer abuse (OR: 1.80; 95% CI 1.26-2.57) and business abuse (OR: 1.75; 95% CI 1.25-2.47) were associated with elevated odds of a positive depression screening score.
Hiott et al, 2008(22), United States 2003	125 farmworkers from Mexico, Guatemala and Honduras	>18	All male	Cross-sectional	MFWSI, PAI, CES-D, CAGE/4M	Regression models	Rigid work conditions including discrimination and abuse by employer	Depression Anxiety Stress	42% of the workers classified as potential clinical depression case, 18% as potential clinical anxiety case, and 38% had a significant level of stress. The more rigid work conditions they had, the higher level of anxiety (β =0 .247 p=0 .005) and depression (β =0 .325 P =0 .000)
Hoppe et al, 2010(65), United states NA	59 non-migrant and 59 Latino warehouse workers	33.3±8.25	3/56	Cross-sectional	NIOSH, GJSQ, GHQ12, Stress-in General Scale	Regression models	Management fairness Wage fairness	Job stress Well- being	Among Latino workers, management fairness was negatively associated with job stress (Adjusted R2=0.35 p<0.001 β= -0.59 p<0.001) No association between wage fairness and psychological well-being among Latino workers (Adjusted R2=0.08 β=0.21 p=0.20)
Karkar et al, 2015(25), Saudi Arabia NA	93 nurses (74 migrants)	NA	All female	Cross- sectional	Modified stress and burnout questionnaire	Pearson’s product-moment correlation	Job insecurity	Stress Burnout	17% of the migrants reported job insecurity, and 8% reported less job compensation as sources of stress. 8% of the migrant nurses reported less job compensation. 76% of the migrants reported different level of stress and70% different levels of burnout.
Kim-Godwin et al, 2004 (66), United States 2002	151 migrants and seasonal workers (Mexican, Cuban, and other)	17-58	NA	Cross- sectional	MFWSI, questionnaire	T-test, ANOVA, Pearson correlation	Job insecurity Low income Undocumented status	Stress	Migrant farm workers reported a higher level of stress in job/legal insecurity compared to the seasonal farmworkers (p<0.05). 51% of the workers perceived high level of stress.
Liu et al., 2020(67), Australia 2014-2015	8969 individuals from a representative sample of Australian households (1731 migrants)	15-64	4424/ 4545	Cross-sectional	3-item scale for job insecurity, MHI-5	Chi-square test, linear regression, Likelihood Ratio test	Job insecurity	Mental health score	Among all participants (i.e., Australian-born and migrants), an increase in job insecurity was associated with a 1-point decrease in the mental health score; no evidence that migrant status acted as effect modifier of the relationship between job characteristics and mental health.
Miller et al, 2005(100), United Kingdom NA	208 migrant teachers from different countries	21-65	160/48	Mixed methods cross-sectional	GHQ-12, RSES, CCS, questionnaire	T-test and multivariate analysis	Ethnic discrimination at workplace Institutional racism	Distress	Over 44% of the participants experienced high levels of distress. Migrant workers were exposed to various types of discrimination (prevalence 11 to 22%) while 48% of them perceived institutional racism at their workplace.
Negi et al, 2019(68), United States 2013-2014	225 Latino horse workers	≥17	32/193	Cross- sectional	CES-10, questionnaire	Bivariate correlation, Multiple regression	Job insecurity Discrimination at work	Depression Work stress	Workers who reported higher job insecurity were more likely to report higher depressive symptoms (β= 0.23, p< 0.001).Significant correlations between job insecurity, depression, and work stress, and between discrimination at work and depression(β=0.26 p<0 .01 B(SE)=0.08(.02)R2=0.18)
Panikkar et al, 2014(69), United States 2006-2009	212 low wage migrant cashiers, cleaners, construction and factory workers from different countries	≥18	105/ 107	Cross-sectional	Survey	Logistic regression	Lack of health insurance	Depression Stress	56% of migrant workers lacked health insurance, and almost 60% of them had psychological hazards.
Robert et al, 2014(70), Spain 2008-2011	318 migrant workers from Colombia, Ecuador, Morocco and Romania	>18	161/ 157	Cohort (2 years of follow-up)	GHQ12, questionnaire	Logistic regression	Undocumented status Lack of health insurance Low income No contract	Poor mental health	Increased risk for poor mental health among individuals with undocumented status (aOR: 17.34; 95% CI 1.96-153.23), lack of contract (aOR:2.24; 95% CI 0.76–6.67), lack of insurance (aOR:2.62; 95% CI 0.62–11.17), and continues low income (aOR:2.73; 95% CI0.98-7.62). Prevalence of poor mental health was 23% among male workers, and was 36% among female workers.
Rosmond et al, 1998(71), Sweden 1992	121 migrant and 711 non-migrant workers	≥48	All men	Cross- sectional	Questionnaire	Crude relative risks by Mantel-Haenszel procedure	Low influence on work situation	Insomnia Melancholy Depression Anxiety	No association between low influence on work situation with insomnia or melancholy among migrants, but low influence on work situation was associated with high degree of melancholy among Swedes. 30.5 % of the employed migrants used medication for psychiatric health problems, 65.5% of them declared insomnia, and 75% of the migrants indicated job stress.
Sidorchuk etal, 2017(72),Sweden 2002, 2006, 2010	43444 non-migrants, 3619 refugees and 4055 non-refugee migrant workers	18-64	3532/ 4142	Cross- sectional	GHQ-12, questionnaire	Chi-square test, logistic regression	Temporary employment	Distress	Migrants who were temporarily employed were more likely to report psychological distress (aOR:1.60; 95% CI 1.34-1.92). The prevalence of psychological distress was 19.8% among Swedish-born migrants, and 26.6% among migrants who were refugees.
Sousa et al, 2010(73), Spain 2008-2009	1.849 foreign-born (Morocco, Ecuador, Romania, and Columbia), and 509 Spanish-born workers	20-39	761/ 1088	Cross- sectional	GHQ-12, questionnaire	Logistic regression including analyses stratified for gender	Undocumented status Temporary contract No contract	Poor mental health	Compared with male permanently contracted non-migrant workers, worse mental health was seen in undocumented migrant workers - who lived in Spain ≤ 3 years (aOR:2.26; CI 1.15-4.42) and who lived in Spain > 3 years and worked with temporary contract (aOR: 1.96; 95% CI 1.13–3.38). 43% migrant women who were in Spain ≤3 years and had no contract experienced poor mental health; 36% of those who had a temporary contract indicated mental health problems. 27% of the migrant men who were in Spain > 3 years and had no contract experienced poor mental health and 31% one of those who had a temporary contract indicated mental health problems.
Sznajder et al., 2022(74), China 2010	696 female factory workers (167 migrants)	18-56	696/0	Cross-sectional	CES-D, Zung Depression Scale, Job Content Questionnaire	Logistic regression	Low job security	Depression Hope-lessness	No differences in perceived job security between migrant and non-migrant workers; 22.9% of the participants indicated depression; No evidence for association between job insecurity or migrant status and depression.
Teixeira et al, 2018(75), Portugal 2009	1322 migrant workers from Brazil, the African Countries	≥18	674/ 648	Cross-sectional	Psychological distress scale, questionnaire	Linear regression	Job insecurity Low income Undocumented status	Distress	Over 21% of the participants reported high psychological distress, over 26% had undocumented status, 57.6% reported low income, and 30.8% reported job insecurity. Job insecurity (p<.001), undocumented status (p<.05), and insufficient income (p<.001) contributed to psychological distress.
Wadsworth et al, 2007(76), United Kingdom NA	626 workers (410 were migrant workers from Africa and Bangladesh)	18-65	180/ 230	Cross- sectional	GHQ-28, Effort–reward imbalance	Chi-square tests and analysis of variance	Racial discrimination Unfair treatment at workplace Temporary contract	Job stress Distress	13% of the migrant workers reported experiencing high job stress, while 22% migrant workers indicated psychological distress. Highest prevalence of work stress among workers with African origin. Racial discrimination (OR: 2.71; 95% CI 1.25–5.90) and unfair treatment (OR; 95% CI 5.74:1.88–17.53) were associated with work-stress among all workers.
Vahabi et al, 2018(26), Canada December 2014 to February 2015	30 Live-in care givers (temporary migrant workers)	25-60	All female	Mixed methods (Cross sectiona)	WHO Well-Being Index, CES-D 20	Bivariate statistics (Chi square, t-test, ANOVA)	Low income Long and unpaid working hours Emotional and physical abuse	Poor mental health Depression Sleep problems	23% of the migrant workers had poor psychological well-being, 30% reported poor mental health, 43% had possibility of major depression, 48% experienced sleep problems at least 2-3 times/week. 73% had low income, and worked more than 40 hours/week. Depression was significantly correlated with the average number of hours worked in a week (p=0.026).
Vives et al, 2013(77), Spain October 2004 and July 2005	5317 non-migrant and 362 migrant manual workers	16-65	154/ 208	Cross- sectional	EPRES. SF-36 Spanish version	ANOVA Pearson chi-square tests	Precarious employment	Poor mental health	Prevalence of poor mental health was higher among migrants compared to non-migrants, and highest among migrant women (33.1% (33.1-47.2). Fully adjusted prevalence of proportional rate (PPRs) of 5th quintile of precarious employment was 2.23 (95% CI 1.77–2.81) in women and 2.18 (95% CI 1.83–2.59) in men.
Vives et al, 2011(78), Spain 2004-2005	6221 non- migrant and 556 migrant workers	16-65	237/ 317	Cross- sectional	EPRES SF-36 Spanish version	Pearson chi-square tests	Precarious employment	Poor mental health	Prevalence of high- to moderate precariousness was 18.3% among migrants. Total precariousness was higher among migrants, with the highest prevalence among young migrant women (88.6%). A significant number of cases of poor mental health was attributable to precariousness among both gender and groups.

The qualitative studies were published in 2003 to 2022, but the underlying data were collected from 1998 to 2019, and most of these papers (73%) were published after 2011 (table 2). Except for one paper, the data from all the qualitative studies were collected in 2006 and afterwards. Semi-structured questionnaire forms were generally used for data collection in focus group discussions or individual interviews. Purposive sampling was the preferred data collection method.

**Table 2:** Characteristics of the qualitative studies. [NA=not available.]

Author, year, country (reference) Data years	Study population (country or nationality)	Age	Gender (F/M)	Study design	Measurement(s)	Statistical analysis	Main themes/categories
Alemi, 2018 (24), Turkey 2015	15 undocumented male Afghan migrants	17-37	All men	Qualitative	Semi-structured interviews	Qualitative content analysis	Themes: (1) motives for migrating to Turkey; (2) traumatic transit experiences; (3) life difficulties in Turkey; and (4) hopes for the future Categories: (a) pre-migration stressors; (b) transit-related experiences’ (c) post-migration stressors; and (d) future desires’ while emergent subcategories included ‘economic stress’, ‘witnessing atrocities’, ‘poverty and unemployment’, and education.
Ahonen, 2009(43), Spain 2006-2007	158 documented or undocumented migrant workers from Colombia, Morocco, Sub-Saharan Africa, Romania, and Ecuador	18-60	68/90	Qualitative, exploratory, descriptive	Semi-structured focus groups and individual interviews, audio-recorded criterion sampling	Narrative content analysis	Themes: (1) overview of working conditions; (b) working conditions and hazards; (c) formal hazard prevention; (d) ‘‘papers’’, migrant status and ‘‘no choice’’
Agudelo-Suarez et al, 2009 (37), Spain 2006-2007	158 migrants from Romania, Morocco, Ecuadoria, Columbia, and Sub-Saharan Africa	18-60	68/90	Qualitative, descriptive, and exploratory	Semi-structured interviews, and focus group, snowballing methods, audio-recorded	Narrative content analysis	Themes: (1) concept of discrimination amongst migrant people; (2) discrimination from a social and political perspective; (3) discrimination, employment and working conditions; (4) the impact of discrimination on mental health and health services access; and (5) protective factors against discrimination
Agudelo-Suarez et al, 2022 (97), Colombia 2018	31 Venezualan migrant workers, 12 informats	>18	NA	Qualitative study	Semi-structured interviews, snowballing methods, theoretical and/or intentional sampling, audio record	Narrative analysis	Themes: (1) the migratory process: reach and difficulties (2) Conditions of work and employment (3) Situation of health and access to services (4) Situation of health and access to services (5) Expectations and plans for the future
Cain, 2021 (102), Australia 2015-2017	30 migrant workers from Afghanistan, Iraq and South Sudan	18-55	11/19	Mixed methods	The open-ended interview questions Semi-structured face to face interview Audio record	Content analysis	Themes: (1) Underemployment (2) Precarious work (3) Financial pressure (4) Unfair treatment (5) Positive aspect of work
Chavez, 2017 (98), Mexico 2012, 2013, 2014	40 unauthorized returned migrant workers from Mexico who worked as a roofer in the US	Mean: 35 years	All men	Qualitative	In-depth interviews, snowball sampling	Qualitative analysis	Themes: (1) social organization of roofing; (2) employment insecurity and social isolation; (3) occupational risks and returning injured; (4) death and the trauma of being a roofer
Carlos et al, 2018 (99), Canada 2006	21 migrant Filipina caregivers	20-69	All female	Qualitative	Semi-structured interviews	Qualitative data analysis	Themes: (1) reasons for choosing to work in Canada (2) perceptions of health; (3) employment and health; and (4) accessibility to healthcare services. Categories: (a) work responsibilities; (b) long work hours; (c) living-in; and (d) separation from family.
Dean, 2009 (83), Canada NA	22 migrants from India, Pakistan, Iraq, and other	25-59	6/16	Qualitative, exploratory	In-depth interviews Semi-structured design, audio-recorded, questionnaire (sociodemographic characteristics and use of other services)	Qualitative analysis based on grounded theory approach	Themes: (1) mental health impacts; (2) physical health impacts .
Eggerth et al, 2019 (41), United States NA	77 Latino workers working as cleaners.	19-80	59/18	Qualitative, exploratory	Semi-structured questionnaire, focus group interviews Snowball sampling	Qualitative data analysis based on using the grounded theory approach	Themes: (1) economic vulnerability; (2) excessive workload; (3) psychosocial stressors; (4) health and safety effects; Categories: (a) precarious work; (b) unpaid/delayed wages; (c) family impact; (d) stress related to management practices; (e) social stigma/dehumanization; (f) peer-group network; (g) chemical hazards; (h) ergonomic hazards; (m) normalization of injury; (n) inconsistent self-appraisal of health.
Fleming et al, 2017(88), United States 2013-2014	34 Latino male migrants, working as day labourers	26-52	All male	Qualitative, exploratory	Semi-structured interviews, focus groups Community mapping and photo voice methods	Thematic content analysis	Themes: (1) marginalisation and discrimination, and its links to health outcomes Categories: (a) employment and occupational illness/injury; (b) stress, anxiety and depression; and (c) limited access to healthcare.
Galon et al, 2014 (89), Spain 2012	44 workers from Colombia, Ecuador, and Morocco	30-51	22/22	Qualitative, exploratory	Focus groups Theoretical and snowball sampling	Qualitative content analysis	Themes: (1) factors associated with presenteeism; and (2) health conditions associated with presenteeism Categories: (a) poor employment conditions; (b) fear of unemployment; (c) the employer/employee relationship; and (d) difficulties in finding temporary replacement workers.
Hall, 2019 (34), China 2015	22 temporary female Filipino domestic workers in Macao, China, and 7 key informants	Mean 42.9±7.4	All female	Qualitative	Focus groups (audio recorded), purposive sampling	Qualitative content analysis	Themes/categories: (1) key health problems identified: (a) poor physical health; (b) poor mental health; (c) non-specific medical problems; (d) stress and included chronic body pain, dizziness, loss of consciousness, and extreme fatigue. (2) determinants of health: social and community networks social relationships; (3) determinants of health: living and working conditions: (a) work environment; (b) healthcare services; and (c) housing (4) social determinants of health: general policy and cultural environment: (a) inadequate labour protection; (b) perceived discrimination (5) social determinants of health: social and community networks
Hsieh, 2016 (38), United States NA	27 Latina hotel housekeepers from Mexico, El Salvador, Honduras, and Guatemala	22-52	21/6	Mixed methods (Qualitative A small-scale ethnography study)	Semi-structured in-depth interview guides, hotels were randomly selected	Qualitative analysis	Themes: (1) personal background; (2) overall work experiences; (3) physical work conditions; (4) equipment and supplies; (5) job satisfaction, job security, supervisor/co-worker support, and work stress; (6) health and safety in the workplace; (7) physical and mental health; (8) methods of dealing with workplace injuries/illnesses and stress.
Labonté et al, 2015 (90), Canada 2009-2011	147 participants, and 117 of them were migrants	>18	NA	Qualitative	Semi-structured interview, digitally or manually recorded, snowball sampling	Thematic analysis and constant comparative methods	Themes/categories: (1) experience of the three major globalization-related pathways: (a) labour markets; (b) housing markets, and social protection programs; and (c) government social protection policies; (4) impacts (of the pathways) on health, standard of living, and future expectations.
Leon-Perez, 2021 (91), United States 2014	30 Mexican immigrant women	>18	All female	Qualitative study	Focus groups, semi-structured interview, audio-recorded	Qualitative analysis based on inductive and deductive analysis	Themes: (1): “Now Mom has to Work 100%”: Work as a Central Source of Stress (2) Parenting Stress in the Context of Precarious Work
Martínez et al, 2015 (92), United States 2011	18 Latin migrant day labourers from Brazil, Ecuador, El Salvador, Guatemala, Honduras, Mexico, and 9 key informants from Colombia, El Salvador, Guatemala, Venezuela, and United States	20-48	10/8	Qualitative	Semi-structured interviews, focus groups, digitally recorded, brief demographic questionnaire Community-based participatory research (CBPR) method	Thematic analysis	Themes/categories: (1) the potential dangers at work that reflect psychosocial stressors for Latina/o migrant day labourers are: (a) anxiety to beat the deadline; (b) fear of wage theft and sudden termination; and (c) the fear of immigration enforcement at the workplace.
Magaña, 2003 (35), United States 1998	75 migrant farmworkers of Mexican descent	16-65	38/37	Mixed methods	Exploratory qualitative interview, audio-recorded	Qualitative analysis	Themes/categories: (1) migrant farmworker stressors; (2) being away from family and friends; (3) rigid work demands; (4) unpredictable work/housing and uprooting.
Nilvarangkul, 2010 (93), Thailans NA	70 Laotian migrant workers working in small-scale cotton mattress production facility, rice mills, slaughterhouse, noodle making factory, lumber mills, and nightlife venues	NA	NA	Qualitative	Participant observation, in-depth interviews, audio recordings, and field notes, purpose sampling and action research methodology based on the concept of Lewin.	Qualitative content analysis	Themes/categories: (1) the issues that caused stress are; (a) living with poverty; (b) non-standard wages and having limited choices (c) loneliness; (d) abuse by employers and local people; (e) distrusting their spouses’ competition in the workplace and job uncertainty; and (f) invisible persons.
Panikkar, 2015 (94), United States 2007-2008	8 migrant women workers, employed in informal work sectors such as cleaning, and factory work from Brazil, Colombia, and Honduras. 8 community key informants	30-52	All female	Qualitative	Semi-structured, conversational style in-depth interviews, audio recorded	Systematic hierarchical thematic analysis	Themes: (1) low family income/living in poverty/receiving poor pay; (2) anxiety and depression; (3) the relationship of migrant farmworker stressors to anxiety and depression Categories: (a) poor housing conditions; (b) language barriers; (c) educational stressors; (d) hard physical labour; (e) lack of transportation and unreliable transportation; (f) exploitation; and (g) lack of day care.
Porthé et al, 2009 (44), Spain 2006-2007	44 undocumented migrants in Spain from Colombia, Ecuador, Morocco, and Romania	18-55	21/23	Qualitative	Focus groups and personal interviews	Qualitative analysis	Themes: (1) instability; (2) empowerment; (3) vulnerability; (4) salary level; (5) social benefits and the ability to exercise rights; (6) working time; (7) health problems related to the work situation;
Premji et al, 2018 (95), Canada 2014	27 migrant workers from Bangladesh, China, Egypt, Mexico, and Other	21-60	15/12	Qualitative	In-depth and semi-structured interviews, audio recorded, participants were recruited using posters, peer researcher networks, and partner agencies.	Qualitative analysis based on inductive method	Themes: (1) participants’ labour market experiences; (2) pathways and mechanisms between precarious employment and health and well-being Categories: (a) stress; (b) material and social deprivation; (c) exposure to hazards; (d) difficult commutes; and (e) childcare challenges.
Premji et al, 2017(96), Canada NA	30 female workers from Afghanistan, Iran, China, Burma, Philippines, Bangladesh, Nepal, Pakistan, Somalia, Sudan, Sierra Leone, Mexico, Uruguay, and Albania	30-59	All female	Qualitative	In-depth interviews snowball sampling, audio recorded Recruitment through posted flyers, partner agencies and peer researcher networks, and snowball sampling	Thematic analyses based on a community-based participatory action research model	Themes: (1) participants’ labour market experiences; (2) pathways between under/unemployment and health
Romero, 2018(39), United States 2015	61 non-union front-of-house workers, for example, hosts, cashiers, servers, bartenders, runners, bellhops, guest room attendants, porters; and kitchen workers, 47 of them Asian/Pacific Islander and Hispanic/Latino migrant workers	29-77	NA	Mixed method	Focus group discussion, digitally recorded Theoretical sampling	Qualitative analysis using grounded theory	Themes/Categories: (1) employee work activities and exposures (level 4). Its categories: (a) employee health at risk (work activities, work-based exposures, and barriers to protection or safety); (b) employee health compromised (injuries, chronic pain/health issues/working sick, and coping strategies: personal (self-treatment) and job-based (documentation) (2) employee job vulnerability (level 3). Its categories: (a) reprimands/warnings; and reluctance to make reports (3) employer-controlled factors (level 2). Its categories: (a) worksite-based (lack of training/understaffing, supplies/equipment needed for job, and work/shift/task assignments) (b) management’s subjective interaction with employees and policy non-compliance (leave policies (illness, vacations, breaks), worker discrimination/favouritism and harassment) (4) lack of management concern for employees (level 1)
Ronda et al, 2016(86), Spain 2012	44 migrant workers from Colombia, Ecuador and Morocco	31-52	22-22	Qualitative	Focus group discussion, audio-recorded, theoretical sampling	Qualitative analysis using grounded theory	Themes/categories: (1) migrant workers’ experiences prior to the crisis: progressive integration in the labour market; (2) employment consequences of the crisis: its categories: (a) worsening of working conditions; and (b) reduced occupational health and safety protection (2) individual consequences of the crisis: its categories: (a) negative effects on health; and (b) effects on family relationships and reduced access to recreation and leisure
Snipes et al, 2007(101), United States NA	69 Mexican migrant farmworkers	≥18	35/24	Mixed method	Focus-group interviews, audio-recorded	Qualitative analysis	Themes: (1) perceptions of stress; (2) Mexican migrant farmworker stress; (3) family-related stress; and (4) living in a different culture
Tang et al, 2017(87), United Kingdom NA	22 Chinese service users in the UK, having received a psychiatric diagnosis	>18	19/9	Qualitative	In-depth life history interviews, purpose sampling	Thematic a nalysis with constant comparative method	Themes: (1) labour market and work conditions; (2) marriage and family; (3) education; (4) aging
Weishaar et al, 2008(32), Scotland 2007	15 Polish migrant workers who work in manual and low-skilled jobs	17-51	9/6	Qualitative	Eight in-depth interviews and two focus groups, digitally recorded	Thematic analysis	Themes: (1) difficulties with communication; (2) unfamiliarity with culture and society; (3) work-related stress; (3) practical stress; (4) social stress; (5) health
Winkelman et al, 2013(84), United States 2008	29 Latino migrant and seasonal farmworkers	18-83	15/14	Mixed methods	Semi-structured questionnaire, focus groups	Thematic analysis	Themes/categories: (1) physical stress related to working conditions; (2) mental stress related to family situations, work environment, documentation status, and the lack of resources: its categories: (a) related to family situations; (b) related to work environment; (c) related to documentation status; (d) related to the lack of resources (3) depression related to separation from family and the lack of resources; (4) use of positive and negative mechanisms for coping with stress and depression.
Vahabi et al, 2017(42), Canada 2014-2015	30 Live-in care givers migrant workers from Philippines, and the rest from Eastern Europe (i.e.Hungary, Ukraine and Poland)	25-60	All female	Mixed method	Self-completed questionnaires and focus groups, audio-recorded	Inductive thematic analysis	Themes: (1) working-and-living conditions; (2) substandard working conditions; (3) being “captive labourers”; (4) housed but homeless; (5) caught between a rock and a hard place; (6) stress, health decline and social support; (7) mental health, resilience and access to care Categories: (a) stress related to work demands; (b) stress related to loss and grief; (c) ambiguous role of family as social support; (d) social support beyond family; (e) perspectives on mental health; (f) perspectives on mental illness; and (g) access to mental health services.
Ziersch et al, 2021(85), Australia. 2018-2019	30 migrants from India, Pakistan, China, Iran, Afghanistan, Vietnam, Sri Lanka, and Sudan	18-55	9/21	Qualitative	face-to-face, semi-structured, in-depth interviews	Thematic analysis based on five-stage framework approach	Themes: (1) Employment Experience in Australia; (2) Experiences of Exploitation/Discrimination; (3) Taking Action in Response to Exploitation and Discrimination

### Prevalence of precarious employment and mental health problems

The majority of the quantitative studies reported more than one precarious work condition; 26 reported the prevalence of precarious employment and 28 provided an estimate for the prevalence of mental health problems among their participants. In some quantitative studies, the majority of participants were exposed to one of the dimensions of precarious work: non-permanent contracts (84%) (82), low-income (73%) (37), lack of health insurance (58%) (82), unfair treatment (54%) and job insecurity (63%) (57), precariousness (67%) (80).

Seven themes emerged from the codes of the qualitative studies: mental health and six other themes representing dimensions of precarious employment. All precarious work conditions and mental health problems reported in the quantitative studies likewise fit into the investigated themes. However, three quantitative studies measuring precarious employment characteristics over the EPRES total score were also included in each theme as they covered all identified themes in this review. The frequency of the themes created by combining quantitative and qualitative data was as follows: mental health (all qualitative and quantitative studies), temporariness (65%; 19 quantitative, 23 qualitative), vulnerability (58%; 13 quantitative, 25 qualitative), imbalanced interpersonal power relations (52%; 19 quantitative, 15 qualitative), disempowerment (46%; 9 quantitative, 21 qualitative), lacking workers’ rights (51%; 12 quantitative, 21 qualitative) and low income (35%; 12 quantitative, 13 qualitative). The included studies reported various mental health problems, most commonly stress (52%), depression (43%), anxiety (34%), poor mental health (23%) and sleep problems (23%).

### Precarious employment and its association with mental health

Some quantitative studies provided just the frequency of exposure, while others presented estimates for the association between exposure and mental health. The main dimensions of precarious employment and their associations with mental health problems are summarized in table 1. Twenty-three quantitative studies reported effect estimates that provided evidence for an association between the respective dimension of precariousness and poor mental health (figure 2).

**Figure 2a:**
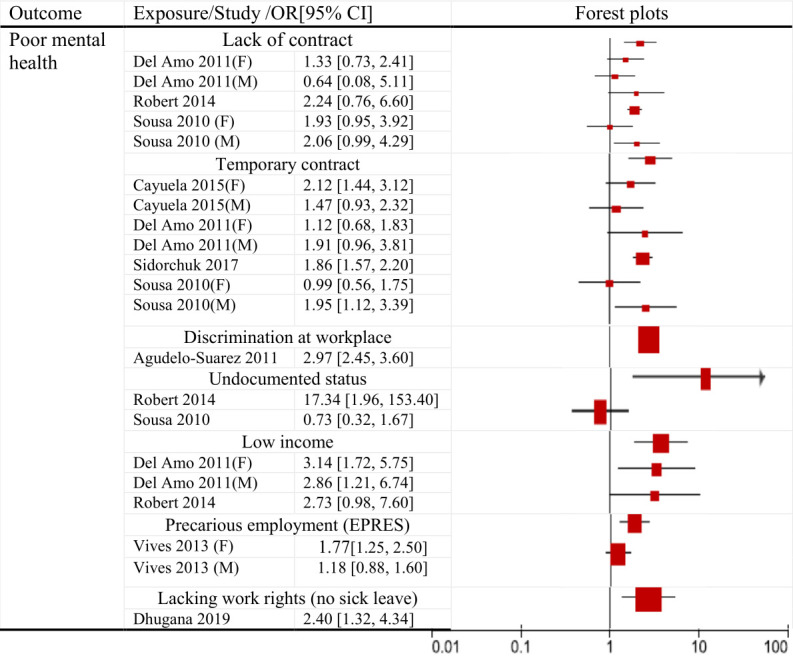
Effect estimates and cofidence intervals (CI) from qualitative studies fod the association between the respectibe exposure (dimension of precarious work) and poor mental health.

**Figure 2b:**
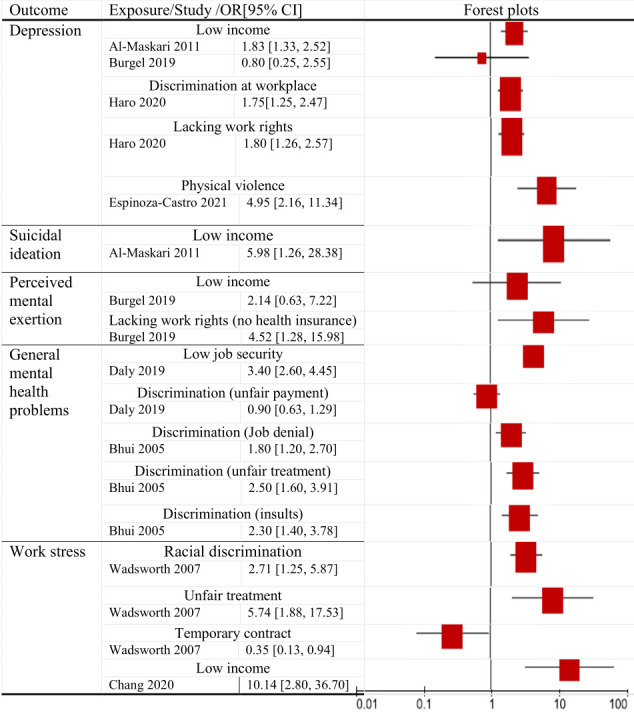
Effect estimates and cofidence intervals (CI) from qualitative studies fod the association between the respectibe exposure (dimension of precarious work) and depression, suicidal ideation, perceived mental exertion, general mental health problems and work stress.

In total, 341 codes were identified from the qualitative studies. Seven themes and 44 categories emerged. Since these identified themes also included precarious work conditions defined in the quantitative studies, the findings regarding precarious employment and its association with mental health problems were given below based on the themes (figure 3). More detailed information about the themes, categories and quotes that best represented these themes and categories are provided in supplementary table S4.

*Theme 1:* Mental health problems. Mental health problems were apparent in all the included studies. Stress was reported in all but six of the qualitative studies (24, 35, 43, 85, 89, 102). Anxiety was reported in 18 qualitative studies (32, 34, 35, 37, 41–44, 83, 86, 88–90, 92, 95–97, 102), depression in 16 (32, 34, 35, 37, 42, 83–86, 88, 89, 94–97, 99) and sleep problems in 12 (32, 34, 39, 42–44, 84, 85, 95–97, 99). Precarious working conditions were found to be important factors underlying workers’ mental health problems. The most common underlined factors were job insecurity (41–43, 83–86, 88–92, 94, 95, 97, 98, 101, 102), low income (32, 35, 38, 42, 84, 86, 89, 93, 95, 96), bad and disrespectful behavior of employers (39, 42, 44, 85, 87, 93, 95, 96), job and income uncertainty (26, 83, 85), undocumented status (42–44, 84, 88, 98) and fear of deportation (42, 43, 88, 101), long working hours (41, 84, 95), need for multiple jobs (90, 95, 96) and discrimination (37, 85, 101).

*Theme 2: Vulnerability*. Vulnerability was the most apparent theme, appearing in 83% of the qualitative studies (24, 32, 34, 37–39, 41–44, 85–87, 89–97, 99, 101, 102). This theme consisted of six categories: fearing termination for insubordination, being unable to ask for better work conditions, being conditioned to feel easily replaceable, working under conditions inconsistent with their contracts, being paid less than are non-migrants and discrimination and racism at workplace. The most evident category within this theme was “fearing termination for insubordination”, which was present in more than one third of the included qualitative studies. It consisted of four subcategories: feeling obliged to work during sickness, receiving insufficient overtime pay despite being requested to render overtime work, being requested to perform additional work or tasks without added payment, working on assigned tasks or jobs without prior consultation notice to the work (24, 32, 34, 37–39, 41–44, 85–87, 89, 90, 92, 93, 95, 97, 102). Being undocumented further worsened these working conditions (24, 37, 85, 102). The studied workers felt too vulnerable to use their rights when needed due to their fear of termination. Thus, many of them went to work despite experiencing work accidents or being in strong pain. Some of them hid their health problems or any work accident/injury to protect their work positions, as some employers prefer not to work with workers who are sick or were in accidents (34, 41). Some workers were exposed to retaliation and threats when they complained about their work conditions (95). Many of the participants expressed that they had accepted their precarious work conditions to survive (86). Furthermore, the inability to ask for better work conditions was common among the undocumented workers (24, 43, 85, 102). Additionally, many participants were aware of their contractual rights but felt too vulnerable to struggle for them (41, 44, 85, 86, 89, 95, 101). Overall, the undocumented migrant workers were more vulnerable compared with the registered migrant workers. Thirteen quantitative studies reported discrimination at the workplace (21, 22, 57, 60, 62–65, 68, 76, 79, 81, 100), and ten of them showed a statistically significant association between discrimination/unfair treatment and mental health problems


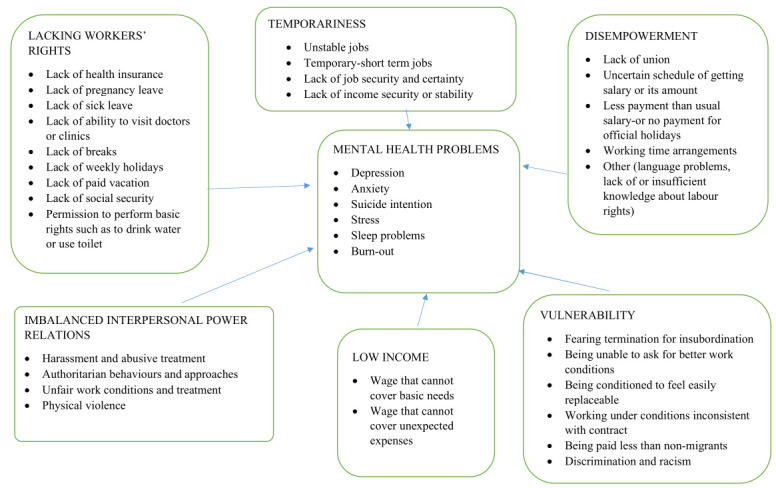


*Theme 3: Temporariness*. This theme emerged in 88% of the qualitative papers. It covered four categories: unstable jobs, temporary/short-term jobs, lack of job security and certainty and lack of income security or stability (32, 34, 38, 41, 42, 44, 83–86, 88–98, 101, 102). Temporary jobs and instability prevent workers from obtaining unemployment benefits (43, 90). Some of them found work through informal resources (44), temporary placement agencies (95) or meeting points in cities where employers recruit workers daily (44). Many of them worked on short-term contracts or without any contract, leading to job inconsistency (44, 83–86, 90, 95, 101), economic instability and lack of employee rights (101). Job insecurity was very common, regardless of the type of precarious contract (32, 38, 89, 90, 93, 98).

Nine quantitative studies (25, 33, 57, 66–68, 74, 75, 80) reported job insecurity, and five of them provided the prevalence of job insecurity (25, 57, 67, 74, 75). Seven studies reported an association between job insecurity and mental health problems (33, 57, 66–68, 75, 80). Many of the studied migrants in the quantitative studies worked on fixed-term, temporary or verbal contracts or no contracts at all (40, 57, 58, 72, 73, 76, 82). Six of the quantitative studies reported an association between precarious contract types and poor mental health (58, 60, 72, 73, 76, 82) and three of them provided gender-stratified analyses. In one of them, migrant women workers reported mental health problems more often compared to non-migrant women workers, even though this difference was not statistically significant (82). Undocumented male migrant workers who worked with temporary contracts reported a higher risk of mental health problems compared to the non-migrant men who worked with a permanent contract (73). In another study, the association between precarious employment and mental health was stronger among women workers than among men (77).

*Theme 4: Lacking workers’ rights*. This theme emerged in more than two thirds of the qualitative studies (24, 32, 34, 35, 37–39, 41, 42, 44, 85, 87, 89, 94–99, 101, 102). Workers who worked on an hourly basis did not enjoy the benefits of labor rights, such as health insurance, paid vacation or paid sick leave; this applied to the undocumented workers as well (34, 37, 38, 44, 89, 94, 96, 98). Some of the workers were not able to take breaks for basic needs; some were not allowed to go to the lavatory without permission, and some received pay deductions for spending ‘more’ time in the lavatory (94).

Seven quantitative studies reported workers’ rights (21, 23, 40, 59, 64, 69) with three of them yielding evidence for an association between lack workers’ rights and mental health outcomes (21, 59, 64).

*Theme 5: Imbalanced interpersonal power relations*. The theme ‘imbalanced interpersonal power relations’ was observed in more than 50% of the qualitative studies and consisted of four categories: harassment and abusive treatment, authoritarian behaviors and approaches, unfair work conditions and treatment, and physical violence (34, 35, 37–39, 41, 42, 44, 85, 87, 93, 95, 96, 102). The majority of the workers experienced unfair treatment and conditions at the workplace (38, 39, 42, 44, 85). Disrespectful behaviors and outright abuse from employers, such as yelling (42), scolding and shouting (34, 85, 102), bullying (87) and ignoring of questions (96), were also experienced. Some workers were hit by their employers (87, 93) or their employers’ children (42), scorched with an iron or made to drink soap at the workplace (34).

Six quantitative studies (40, 62, 66, 70, 73, 75) reported undocumented status. Three of them reported an association between undocumented status and poor mental health (62, 70, 73).

*Theme 6: Low income*. This theme contained the categories “wage that cannot cover basic needs” and “wage that cannot cover unexpected expenses” (24, 35, 43, 44, 83, 84, 86, 91, 93, 95–97, 102). Many workers experienced various consequences of earning low income, such as worsening quality and quantity of food (35, 83, 93, 95), inability to provide extracurricular activities for their children [eg, birthday celebrations (95)] and inability to purchase medicine for children (93). Some of them could not pay their bills and other necessities (84), such as transportation, clothing for family members (83, 95) and other types of required expenses (35, 84, 86, 93).

Nine of the included quantitative studies reported low income among the participants, with six of them providing its prevalence (21, 23, 26, 56, 58–60, 66, 70). Four of the studies observed evidence for an association between low income and mental health problems (23, 56, 58, 60).

*Theme 7: Disempowerment*. This theme was apparent in 63% of the qualitative studies covering the following categories: lack of unions, uncertain pay amount or schedule, unusually small salary amount or lack of payment during official holidays, working time arrangements, and other issues (eg, language barriers and insufficient or non-existent knowledge about labor rights) (24, 32, 34, 38, 41–44, 83, 85, 89, 91–96, 99, 101). The majority of the workers had no unions (43, 89) and/or had insufficient information about access to labor unions or any work organization that supports workers. With no official documentation and language problems, the participating workers felt helpless relative to their employers (24, 32, 43, 101). Thus, they were required to work overtime or on public holidays without any payment (38), paying low salaries (24, 32), giving no payment at all (92) or paying wages at uncertain times (34, 93). Some undocumented workers who fought for payment for days worked were threatened by their employers with calls to police, which might lead to deportation (24, 32).

## Discussion

The main purpose of this review of qualitative and quantitative studies was to summarize and analyze existing research on the association between precarious employment and mental health of migrant workers. The review showed that the included studies examined different dimensions of precarious employment and a variety of mental health problems. Some of them reported the prevalence of precarious employment and mental health problems and/or an association between precarious employment and mental health problems. A considerable number of those studies did observe evidence for such an association.

In total, the review included 65 studies from four continents, with almost 90% of the studies being performed in high-income countries, which have had the largest numbers of migrant workers in the last century (3). Most of the included studies were of high quality. The data of almost all the studies were collected and published after the 2000s, and the number of published studies has increased especially in recent years. This development is consistent with the rising prevalence of precarious work conditions in the last decades due to globalization and decline of social policies (5, 9). Mounting awareness of precarious employment and its influence on health and life quality of workers among the general public may also have contributed to the rise in the number of studies in this field.

The results of the present review indicate that a high prevalence of precarious employment among migrant workers with migrants being frequently exposed to various dimensions of precarious employment. On top of this, the results from those studies that also included non-migrant workers indicate that the prevalence of precarious employment tends to be higher among migrant compared to non-migrant workers while at the same time the effects of precarious work conditions on mental health also seem to be more pronounced among migrants (77, 81, 82). Those findings support the assumption of a special vulnerability to precarious employment among migrants outlined at the beginning.

Temporariness, (temporary, fixed-term or verbal contracts or no contracts at all), was the most commonly reported dimension of precarious employment among the included studies. This is in line with the results of related reviews that also concluded that temporariness is the most commonly observed dimension of precarious employment among workers including migrants (5, 19, 27, 29). Furthermore, migrant workers in the studies included in our review commonly expressed a lack of rights or perceived powerlessness to exercise workers’ rights, income insufficiency for basic needs, income insecurity or perceived powerlessness to negotiate work schedules or salaries. For example, two qualitative studies reported that women workers could not even use pregnancy leave and were forced to work until the last days of pregnancy (44, 89). Some studies reported that migrant workers were exposed to highly unacceptable behaviors, such as racism, drinking of soap and scolding with physical violence (34, 42, 87, 93).

In terms of mental health problems, the most commonly investigated ones were stress, depression, anxiety, poor general mental health and sleep problems. The prevalence of these outcomes varied between 10–75% among the quantitative studies included in the present review. Some of the included studies provided data about a putative association between precarious employment and mental health problems, yielding prevailing evidence for such an association. For example, temporariness was a risk factor for mental health problems in most of the studies examining this dimension of precariousness. The influence of this dimension on mental health (2, 5, 29, 30) and occupational accidents and injuries of workers (19, 20) has been widely examined by other systematic reviews, and their results are similar to those of this review. Moreover, the migrant workers found exposure to discrimination and authoritarian behaviors to be hurtful experiences and are thus important factors of mental health. Reviews by Sterud (31) and Jurado (103) also supported this finding as they found a relationship between perceived discrimination and poor mental health among migrant workers. Furthermore, the results of qualitative studies show that workers’ interpretation of precarious work conditions also seems to play an important role in developing mental health problems. Cultural background, education level, professional work experience in original country, perspective of life and workers’ rights, awareness of the occupational system, and rights in the host country may affect their interpretation. For example, workers’ interpretation and acceptance of precarious work conditions as a permanent endeavor or a temporary period may affect the direct relationship between precarious employment and mental health. Some workers resort to precarious employment because of urgent economic needs or a need to obtain a positive reference from an employer, which is required in finding new work or securing a work contract that will extend their residency in the host country. Thus, they may feel the need to endure precarious work conditions, such as discrimination, long working hours without compensation, unpredictable work schedules, lack of workers’ rights and authoritarian or disrespectful behaviors at the workplace. These experiences and feelings of workers may have added impact on their development of mental health problems. Having low social support and family concerns, limited access to and information about health care systems and traumatic life experiences prior to migration may also increase workers’ levels of mental health problems (103).

The findings of this review also provide scientific data on precarious employment and its influence on mental health based on differences in gender and type of migrant status. Being undocumented in the host country seemed to be the worst condition, and it exposed workers to combined dimensions of precarious employment (24, 41, 43). In addition, female migrant workers developed higher rates of mental problems due to exposure to precarious employment compared with male migrant and non-migrant workers (73, 82).

### Strengths and limitations

*Search strategy and publication bias*. This review is the first to provide scientifically comprehensive data about the association between precarious employment and migrant workers’ mental health. Wide-ranging definitions of and approaches to precarious employment, migration, and mental health problems were adopted to identify all relevant studies in the field. In addition to three digital databases, related informal sources and the references of the initially included studies were manually searched to decrease the possibility of overlooking relevant studies. This search strategy covered all relevant observational studies that were published in four languages (English, Spanish, German and Turkish) in the last 50 years. This search produced a reasonable number of qualitative and quantitative studies published in English and Spanish.

A limitation of our review is that no meta-analysis was conducted due to variations between the precarious employment dimensions, mental outcomes and measurement methods in the included studies. For this reason, it was also not feasible to construct funnel plots for assessment. However, the findings from another systematic review suggest that publication bias does not seem to play a major role in the research area of precarious work (5).

Moreover, some sources of bias in the individual studies need to be taken into account. First, most of the quantitative works were cross-sectional studies using different measurement tools to evaluate multiple types of precarious employment and various mental health outcomes. Only three studies assessed precarious employment with a validated and reliable tool (EPRES); the rest used different forms of questionnaires hampering the comparability between those studies. Furthermore, also mental health was measured with different scales or questionnaires. For example, the 12-item general health questionnaire (GHQ-12) was widely used to measure mental health; however, some authors interpreted the level of mental health over the total score (linear outcome variable), whereas some used a cut-off to define a dichotomous outcome variable. Furthermore, there was a lack of data regarding the duration of exposure to precarious employment, whether the workers had any mental health problems before beginning to work and work experiences prior to migration. Finally, limited data on the causes of migration was provided in the included studies, which might have an influence on the development of mental health problems.

### Implications

The results of this review underline the importance of preventing mental health problems by reducing or mitigating precarious work conditions among migrant workers. Additionally, our results may increase social awareness about precarious employment and its influence on mental health, thereby aiding the establishment of human-based, worker-friendly policies at workplaces. This review might also help protect workers from exploitation by enabling experts to control certain elements in workplaces, which seem likely to have precarious work conditions, such as factors related to workers’ rights. Furthermore, our data may play a role in the ongoing discussion about precarious employment being potentially useful for the planning of long-term preventive programmes. We recommend that future qualitative and quantitative studies holistically examine multiple dimensions of precarious employment and their influence on the mental health and well-being of workers using a multidimensional definition of precariousness. When doing so, studies may especially aim to disentangle the underlying mechanisms by being based on sound theoretical frameworks such as occupational stress models (9). For instance, one may argue that some factors, eg, job insecurity, are not working conditions per se but rather mediators on the pathway between precarious employment conditions and mental health. By applying and operationalizing a priori defined theoretical frameworks, a better understanding of such mechanisms may be obtained. Such analyses may also elucidate to what extent the association between precarious employment and mental health depends on migrants’ individual characteristics and competencies or factors related to the migration process (eg, time since migration, reason for migration) (104). Moreover, future research may identify work-related or individual factors that act as job resources mitigating the effects of precarious employment in terms of the Job Demands–Resources model (105). Concerning study designs, most studies included in our review were cross-sectional. Longitudinal studies that can establish a temporal relationship between exposure and outcome would thus be useful in the future.

### Concluding remarks

The results of the present review indicate that migrant workers are exposed to various dimensions of precarious employment and frequently experience mental health problems. An association between different dimensions of precarious employment, most prominently temporariness, and mental problems was reported by a considerable proportion of the included studies. However, it was observed that the theme of disempowerment and lacking workers’ rights were less frequently examined compared to other themes, especially in quantitative studies. Altogether, those findings support the hypothesis that precarious employment is associated with migrant workers’ mental health. We recommend that future research should better disentangle the underlying mechanisms by being based on sound theoretical frameworks as provided by occupational stress models. Ultimately, the results of the present review may be used as evidence for developing a new policy to resolve precarious employment. In addition, this review may also be used as a guideline for developing a better migrant-friendly policy in the future.

### Conflicts of interest

The authors declare no conflicts of interest. This study received no specific grant from any funding agency. One of the authors (OKO) received a scholarship within the Alexander-von-Humboldt Foundation’s Philipp-Schwartz-Initiative. The scholarship provider played no role in the study design, the collection, analysis and interpretation of the data, the writing of the report, or the decision to submit the paper for publication.

## Supplementary material

Supplementary material
