# Supplementary material for: Precarious employment and migrant workers’ mental health: a systematic review of quantitative and qualitative studies
Source: Scand J Work Environ Health. 2022 Jun 30;48(5):327–50. doi: 10.5271/sjweh.4019 (PMC9527784; doi:10.5271/sjweh.4019)
Supplement: Supplementary material [file SJWEH-48-327-S001.pdf]

Precarious Employment and Migrant Workers' Mental Health: A Systematic Review of Quantitative and Qualitative Studies[1]

by Ozlem Koseoglu Ornek,PhD[2], Julia Waibel, Pia Wullinger, Tobias Weinmann, PhD

1. Supplementary material

2. Correspondence to: Ozlem Koseoglu Ornek Institute and Clinic for Occupational, Social and Environmental Medicine; University Hospital, LMU, Munich, Ziemssenstr. 5, 80336 Munich, Germany (E-mail: ozlem.koseoglu@med.uni-muenchen.de).

Table S1: Search strings based on the PECO Framework

Components	Search Terms
**P**opulation	Emigrants and immigrants[MeSH Term] OR undocumented immigrants[MeSH Term] OR transients and migrants[MeSH Term] OR foreigner* OR foreign worker OR foreign workers OR foreign-born worker OR foreign-born workers OR Immigrant* OR migrant* OR oversea worker OR oversea workers OR immigrant worker OR immigrant workers OR migrant worker OR migrant workers OR emigrant*
**E**xposure	Atypical OR casual OR contingent OR discontinuity OR disempowerment OR flexible hour OR flexible hours OR inconvenient hour OR inconvenient hour OR inadequate wage OR inadequate wages OR insufficient wage OR insufficient wages OR minimum wage OR minimum wages OR low wage OR low wages OR poor wage OR poor wages OR inadequate salary OR insufficient salary OR low salary OR poor salary OR minimum salary OR insecur* OR layoff* OR nonstandard OR non-standard OR outsourc* OR outsourced services[MeSH Term] OR part time OR part-time OR downsizing OR personnel downsizing[MeSH Term] OR precarious OR short-term OR staffing agency OR Temporar* OR unprotected OR work condition OR work conditions OR workplace right OR workplace rights OR informal) AND (employment[MeSH Term] OR work[MeSH Term] OR occupations[MeSH Term] OR workplace [MeSH Term] OR job* OR occupation*
**C**omparators	Terms for Comparators will not be included.
**O**utcomes	Mental Health[MeSH Term] OR mental Disorders[MeSH Term] OR depression[MeSH Term] OR depressive disorder[MeSH Term] OR anxiety[MeSH Term] OR anxiety disorders[MeSH Term] OR dyssomnias[MeSH Term] OR sleep[MeSH Term] OR sleep initiation and maintenance disorders[MeSH Term] OR insomnia OR burnout, psychological[MeSH Term] OR burnout, professional[MeSH Term] OR burn-out OR burnout OR stress OR occupational stress[MeSH Term] OR job stress OR work-related stress

All *MeSH term will be used also as free word term * The word with an asterisk after the root stem used, where is appropriate.*

Table S2: Newcastle-Ottawa Scale Adapted for Cross-Sectional, Cohort and Case Control Studies

Articles	Study design	Selection	Comparability	Outcome	Total Score-Quality
Agudelo-Suarez, 2009 (quantitative)	Cross-sectional	***	-	*	4-Moderate
Agudelo-Suárez, 2011	Cross-sectional	**	-	**	4-Moderate
Al-Maskari, 2011	Cross-sectional	****	**	**	8-High
Burgel, 2019	Cross-sectional	***	**	**	7-High
Benach, 2015	Cross-sectional	***	**	**	7-High
Bhui, 2005	Cohort	****	**	**	8-High
Cayuela, 2015	Cross-sectional	***	**	**	7-High
Chang, 2020	Cross-sectional	***	**	**	7-High
Daly, 2019	Cross-sectional	****	**	**	8-High
Del Amo, 2011	Cross-sectional	***	*	**	6-Moderate
Dhungana, 2019	Cross-sectional	**	**	**	6-Moderate
Drydakis, 2022	Cohort	**	**	**	6-Moderate
Espinoza-Castro, 2021	Cohort	***	**	**	7-High
Font, 2012	Cross-sectional	***	**	**	7-High
Grywacz, 2010	Cohort	**	**	**	6-Moderate
Hammond 2010	Case Control	***	*	**	6-Moderate
Haro, 2020	Cross-sectional	***	**	**	7-High
Hiott, 2008	Cross-sectional	***	*	*	5-Moderate
Hoppe, 2010	Cross-sectional	****	*	**	7-High
Karkar, 2015	Cross-sectional	**	*	*	4-Moderate
Kim-Godwin, 2004	Cross-sectional	***	-	*	4-Moderate
Liu, 2020	Cross-sectional	****	**	**	8-High
Miller, 2005	Cross-sectional (mixed method)	**	-	*	3-Low
Negi, 2019	Cross-sectional	**	**	**	6-Moderate
Panikkar, 2014	Cross-sectional	*	*	**	4-Moderate
Robert, 2014	Cohort	****	**	**	8-High
Rosmond, 1998	Cross-sectional	****	-	**	6-Moderate
Sidorchuk, 2017	Cross-sectional	****	***	**	9-High
Sousa, 2010	Cross-sectional	***	**	**	7-High
Sznajder, 2022	Cross-sectional	**	**	**	6-Moderate
Teixeira, 2018	Cross-sectional	**	**	**	6-Moderate
Vahabi, 2018	Cross-sectional (mixed method)	*	**	*	4-Moderate
Wadsworth, 2007	Cross-sectional	**	**	**	6-Moderate
Vives, 2011	Cross-sectional	****	**	**	8-High
Vives, 2013	Cross-sectional	****	**	**	8-High

NOS-Cross-sectional design: Selection: Maximum 5 stars, Comparability: Maximum 2 stars, Outcome: Maximum 3 stars
NOS-Cohort design: Selection: Maximum 4 stars, Comparability: Maximum 2 stars, Outcome: Maximum 3 stars
NOS-Case-Control design: Maximum 4 stars, Comparability: Maximum 2 stars, Exposure: Maximum 3 stars
Total NOS score: low quality (0–3 points), moderate quality (4–6), high quality (7–9).

Table S3: Quality Assessment of the Qualitative Studies using the Critical Appraisal Skills Programme (CASP)

Study ID	1	2	3	4	5	6	7	8	9	10	Total Score-Quality
Agudelo Suarez 2022(qualitative)	Y	Y	Y	Y	Y	Y	Y	Y	Y	Y	10-high
Agudelo-Suarez, 2009(qualitative)	Y	Y	Y	Y	Y	C	Y	C	C	Y	8.5-high
Alemi, 2018	Y	Y	Y	C	Y	Y	C	C	Y	Y	8.5-high
Cain, 2021*	Y	Y	Y	Y	C	C	Y	C	Y	Y	8.5-high
Carlos 2018	Y	Y	Y	Y	Y	Y	Y	Y	Y	Y	10-high
Chavez, 2017	Y	Y	Y	Y	C	C	C	C	Y	C	7.5-moderate
Eggerth, 2019	Y	Y	Y	Y	Y	Y	Y	Y	Y	Y	10-high
Fleming 2017	Y	Y	Y	Y	Y	Y	Y	Y	Y	Y	10-high
Galon 2014	Y	Y	Y	Y	C	N	Y	Y	Y	Y	8.5-high
Hall 2019	Y	Y	C	Y	Y	C	Y	Y	Y	Y	9-high
Hsieh 2016*	Y	Y	Y	Y	Y	Y	C	Y	Y	Y	9.5-high
Labonte, 2015	Y	Y	Y	Y	Y	N	C	C	Y	C	7.5-moderate
Leon-Perez 2021	Y	Y	Y	Y	C	Y	Y	Y	Y	C	9-high
Martinez, 2015	Y	Y	Y	Y	Y	C	C	C	Y	Y	8.5-high
Nilvarangkul, 2010	Y	Y	C	Y	C	N	Y	C	Y	Y	7.5-moderate
Porthé 2009	Y	Y	Y	Y	Y	Y	Y	Y	Y	Y	10-high
Premji 2017	Y	Y	C	Y	Y	C	Y	Y	Y	Y	9-high
Premji 2018	Y	Y	Y	Y	Y	Y	Y	C	Y	Y	9.5-high
Romero et al 2018*	Y	Y	Y	Y	Y	C	Y	C	Y	Y	9-high
Ronda 2016	Y	Y	Y	Y	Y	N	Y	Y	Y	Y	9-high
Snipes 2007*	Y	Y	Y	Y	C	C	C	C	Y	Y	8-high
Tang 2017	Y	Y	Y	Y	Y	Y	C	Y	Y	Y	9.5-high
Vahabi 2017*	Y	Y	Y	Y	Y	C	Y	Y	Y	Y	9.5-high
Weishaar, 2008	Y	Y	C	Y	C	N	C	C	Y	Y	7.5-moderate
Winkelman, 2013*	Y	Y	C	Y	C	C	C	Y	Y	Y	8-high
Ahonen 2009	Y	Y	Y	Y	Y	N	C	Y	Y	Y	8.5-high
Dean 2009	Y	Y	Y	Y	Y	C	C	Y	Y	Y	9-high
Magaña 2003*	Y	Y	Y	Y	Y	N	C	C	Y	Y	8-high
Panikkar 2015	Y	Y	C	Y	C	C	C	Y	Y	Y	8-high
Ziersch_2021	Y	Y	Y	Y	C	C	Y	Y	Y	Y	9-high

1. Was there a clear statement of the aim of the research?
2. Is a qualitative methodology appropriate?
3. Was the research design appropriate to address the aims of the research?
4. Was the recruitment strategy appropriate to the aims of the research?
5. Was the collected in a way that addresses the research issue?
6. Has the relationship between researcher and participants been adequately considered?
7. Have ethical issues been taken into consideration?
8. Was the data analysis sufficiently rigorous?
9. Is there a clear statement of findings?

10. How valuable is the research?

Y: Yes

C: Can’t tell

N: No

*Mixed method

Table S4: The Themes and Categories of the Included Qualitative Studies

Themes	Categories	Sub-categories	Quotations	Supporting studies
LACKING WORKERS' RIGHTS	• Lack of health insurance<br>• Lack of pregnancy leave<br>• Lack of sick leave<br>• Go to dr or clinic<br>• Lack of breaks<br>• Lack of weekly holidays<br>• Lack of paid vacation<br>• Lack of social security<br>• Lack of other basic needs and rights (WC, water etc.) or on permission to use them.		"*the thing that floors me the most is that none of us, we don't get breaks. They don't even acknowledge that we need a break.*"(1)<br>*"I worked until the last day after giving birth, because I was never told "look, you're pregnant, take a few days, no", so I worked the last day and went out and came back on Friday, Saturday and Sunday, and on Monday I gave birth, so I worked until the last day" (GF, women, Ecuador)*(2)<br>"*I was on medical leave for respiratory problems. I got asthma. I was sick for several days. I was bad (...) I think it was four days of medical leave and I had to get up and go to work*" (Female, Ecuador)(3)<br>*''One time I fell from the ladder and had to go to the doctor. I told them [the farm owners] what happened, and was [told that] they didn't want to give me a job anymore.... I begged them [the farm owners] for my job and they said if I was not going back to the doctor any more, they would give me my job back.''*(4)	The studies(1–21)

LOW INCOME	• The wage does not cover basic needs<br>• The wage is not enough for unexpected expenses		"*I don't have enough money to pay the bills. Makes me feel hopeless and want to cry*."(22)<br>"*I get so worried that my head hurts in thinking about how I am going to save enough money to make the truck payments. The payments are $500. We have to make enough money for the payments. I feel so worried and desperate*. (48 years old women)(8)<br>"*Some people ask me the other day, I don't know for what. Some people ask me, ''what kind of food do you like?'' ...I told them it's not what food I like but what I can afford. If I buy chicken, probably I take out the good stuff and give it to my son. The skins, the fat, the bone, came to my bowel. (30 years old man from Vietnam who live in Canada for 10 years)*(16)	The studies:(2,5,8,16–18,21–27)
TEMPORARINESS	• Unstable jobs<br>• Temporary-short term jobs<br>• Lack of job security and certainty		"*Sometimes I find a job and it only lasts two weeks, then I have another two weeks off. Then I find another [job] for another two weeks or only a few days, and that's how I work most of the year''*(4)<br>*"It's much worse because they don't have to sack you, they just say don't come back tomorrow"*(25)	The studies:(2–4,6,7,10–13,15–19,21,22,24–30)

	• Lack of income security or stability	• job insecurity<br>• Job uncertainty	*We are worried that we may lose our job since our job depends on order and material…in the rainy season, cotton mattress demands very low and the employers do not pay us any money. It is also very difficult to find cotton at the moment since some people use synthetic mattress instead of cotton mattress and not many people plant cotton trees (F,L1). (Nilvarangkul, 2010, P. 4)*<br>*"I have to wait for a call, and I have to be very careful about the calls. Ok, and I call them to say, ''Do you have position for today or something like that?'' ''No, no, no at the moment no. We call you.'' But again I call, ''Ok, what happened today? Do you have a position for today or for tomorrow?'' (54 years, a man from Colombia, used to be a teacher in Colombia) (Premji 2018 P. 5)*	
IMBALANCED INTERPERSONAL POWER RELATIONS	• Harassment and abusive treatment	• sexist and abusive behaviours to women<br>• emotional and physical abuse by employer<br>• Harassment<br>• Abusive treatment	"*Since they abuse, they abuse every day, until you get tired, because they think you are a slave and they can do with you*" (men, Ecuador)(2)<br>" *Regarding harassment, one supervisor told a worker taking leave, Okay, you take more than that, you're going to be in trouble.*"(1)	The studies:(1,2,7–11,13,14,16,17,21,25,27)

			''The bad thing is that… umm… I don't want to call it racism, but I can see a little injustice. There are some African-American housekeepers who are on the phone, and the manager will walk by them and act like nothing; but if it's one of us it's different…a lot of the supervisors are Mexican… they will yell at us and embarrass us in front of everyone…''(10)	
	• Authoritarian behaviours and approaches<br>• Unfair work conditions and treatment<br>• Physical violence		"*My employer yells at me and the kids hit me. You cannot do anything, you cannot do anything. If you yell at them you get fired*"(12)<br>*''My employer is very cruel. One time he hit me since I was tired and wanted to stop working'' (A male worker)*(27)<br>*"Yes, then you'll get scorched with an iron. Right, what they are holding, yes." (female domestic worker"*(7)	
VULNERABILITY	• Being unable to ask for better work conditions<br>• Being conditioned to feel easily replaceable<br>• Working under conditions inconsistent with their contracts		'*My employer pushed me to work until dark but he had never paid me overtime wages*'(27)'<br>*"I work long hours but I just get the minimum wage. It's like you're always overworked and underpaid. And then you're tired on top of it. Can you imagine if I was being paid the right amount? I wouldn't have so much stress*(29).	The studies(1–7,9 –18, 20, 21, 23, 25–27, 29, 31)

	• Being paid less than are non-migrants			
	• Discrimination and racism	• Discrimination at workplace<br>• Racism at workplace	*''(.) they see the immigrants working alongside them as being directly responsible for this phenomenon, so, naturally, there are always instances of xenophobia, not so much racism but rather xenophobia among Spanish workers towards foreigners, some are fairly minor incidents that are not really significant, and others are more major''* [A Man – Colombia](9)	
	• Fearing termination for insubordination	• Feeling obliged to work during sickness,<br>• Receiving insufficient overtime pay despite being requested to render overtime work,<br>• Being requested to perform additional work or tasks without added payment,	*"In this time, I know that there is something happening in my back. I stopped work, working, and I told the supervisor I should go home. I can't continue. Do you know what he told me? He told me, ''If you will go now, I will tell [temporary agency] that you have a problem in your back and they will not bring you here again!'' Nobody cared.* (Babu, 40-year-old man from Egypt)(16)<br>*"This has happened to me in most companies where I've been, because. because I don't have legal status, they take advantage. the bosses, because I'm an illegal immigrant they demand. more of you, they made more demands, force you to*	

		• Working on assigned tasks or jobs without prior consultation notice to the work	*do overtime and then they don't want to pay you the proper hourly rate, they make you pay [Men – Ecuador]*(9)	
DISEMPOWERMENT	• Lack of union<br>• Uncertain schedule of getting salary or its amount<br>• Less payment than usual salary-or no payment for official holidays etc.		"*When I first got here I felt like turning myself in [to the immigration authorities] . . . a friend of mine wanted to kill himself because he worked for a while for someone and did not get paid because he didn't speak the language". "We have no power over these [Turkish] people, after working for them they will call the police on us and tell them that they don't know us*"(5)<br>"*I can't report anything because I don't have an employment contract, I know what my situation is, which I am very aware of those things (...)*" (E, woman, Romania)(2)<br>"*I'm sure that for other people, for sure they'd be paid more, because since we come and we don't have work, well, we work for little money because we have to work, and so we accept badly paid work, because*	The studies(2–7, 10–13, 15–17, 20, 23, 24, 26, 27, 30)

			in the moment that you have papers you can choose…you have your rights and everything…you can't choose, you have to accept where you're accepted". (*undocumented woman*, FG)(23)<br>"*When you give my salary delayed, you don't hear anything from me. 9-10 days after the supposed day of compensation, that's when you give my salary.*"(7)	
	• Working time arrangements<br><br>• Other (language problems, lack of or insufficient knowledge about labour rights)	• Worked at unpleasant shifts/hours/days<br>• Unpredictable working schedule or hours	*Well at that work I find myself with shift work, 24/7 because it's at the airport … All the holidays, all the special moments in the family I found myself working … I got sick, I really got sick. I had a panic attack at the airport and I guess it's due to the stress that I was going through. And then the doctor suggests to me that it was too much for me, all this work and the hours and the shift and nights and that stress because of my family, the problems. I could not get the hours that I wanted to. (Lucia, 54 years old, from Uruguay)*" (Lucia, 54 years old from Uruguay expressed)(17)	

MENTAL HEALTH PROBLEMS	• Depression,<br>• Anxiety,<br>• Suicide intention,<br>• Stress,<br>• Sleep problems,<br>• Burn-out<br>• Other mental health problems		"*I worked for eight years and, as the colleagues say, I had a really bad time because the lady was too depressed, and had anxiety and I was becoming depressed and anxious too. I could not sleep anymore. I had to be taking sleeping pills. I was crying all the time. Just crying*" (Female, Ecuador)(3)<br>"*I feel sleepy, then feel bad that I am not doing anything. I feel depressed. I can't drive." "I don't have enough money to pay the bills. Makes me feel hopeless and want to cry.*"(22)<br>*"I don't sleep. I'm always telling my doctors that I haven't slept well because of so much stress. I need, I don't know if a psychologist or someone to help me because I've lost my sleep*"(1).	The studies(1, 2, 11–14, 16–21, 3, 22, 24–30, 32, 4–10)

References

1. Romero D, Flandrick K, Kordosky J, Vossenas P. On-the-ground health and safety experiences of non-union casino hotel workers: A focus-group study stratified by four occupational groups. Am J Ind Med. 2018;61(11):919–28.

2. Porthé V, Benavides FG, Vázquez ML, Ruiz-Frutos C, García AM, Ahonen E, et al. La precariedad laboral en inmigrantes en situación irregular en España y su relación con la salud (Precarious employment in undocumented immigrants in Spain and its relationship with health). Gac Sanit. 2009;23(Suppl. 1):107–14.

3. Galon T, Briones-Vozmediano E, Agudelo-Suárez AA, Felt EB, Benavides FG, Ronda E. Understanding sickness presenteeism through the experience of immigrant workers in a context of economic crisis. Am J Ind Med. 2014;57(8):950–9.

4. Snipes SA, Thompson B, O'Connor K, Godina R, Ibarra G. Anthropological and psychological merge: Design of a stress measure for Mexican farmworkers. Cult Med Psychiatry. 2007;31(3):359–88.

5. Alemi Q, Montgomery S, Stempel C. A Qualitative Study Exploring the Psychosocial Needs of Male Undocumented Afghan Migrants in Istanbul, Turkey. Societies. 2018;8(2):22.

6. Weishaar HB. Consequences of international migration: A qualitative study on stress among Polish migrant workers in Scotland. Public Health. 2008;122(11):1250–6.

7. Hall BJ, Garabiles MR, Latkin CA. Work life, relationship, and policy determinants of health and well-being among Filipino domestic Workers in China: A qualitative study. BMC Public Health. 2019;19(229):1–14.

8. Magaña CG, Hovey JD. Psychosocial stressors associated with Mexican migrant farmworkers in the midwest United States. J Immigr Health. 2003;5(2):75–86.

9. Agudelo-Suárez A, Gil-González D, Ronda-Pérez E, Porthé V, Paramio-Pérez G, García AM, et al. Discrimination, work and health in immigrant populations in Spain. Soc Sci Med. 2009;68(10):1866–74.

10. Hsieh YC, Apostolopoulos Y, Sönmez S. Work Conditions and Health and Well-Being of Latina Hotel Housekeepers. J Immigr Minor Heal. 2016;18(3):568–81.

11. Eggerth DE, Ortiz B, Keller BM, Flynn MA. Work experiences of Latino building cleaners: An exploratory study. Am J Ind Med. 2019;62(7):600–8.

12. Vahabi M, Wong JPH. Caught between a rock and a hard place: mental health of migrant live-in caregivers in Canada. BMC Public Health. 2017;17(498):1–15.

13. Ziersch A, Walsh M, Due C, Reilly A. Temporary Refugee and Migration Visas in Australia : An Occupational Health and Safety Hazard. 2021;

14. Tang L, Pilgrim D. Intersectionality, mental health and Chinese people in the UK: A qualitative exploration. Ment Heal Rev J. 2017;22(4):289–99.

15. Panikkar B, Brugge D, Gute DM, Hyatt RR. “They see us as machines:” The experience of recent immigrant women in the low wage informal labor sector. PLoS One. 2015;10(11):e0142686.

16. Premji S. “It’s Totally Destroyed Our Life”: Exploring the Pathways and Mechanisms Between Precarious Employment and Health and Well-being Among Immigrant Men and Women in Toronto. Int J Heal Serv. 2018;48(1):106–27.

17. Premji S, Shakya Y. Pathways between under/unemployment and health among racialized immigrant women in Toronto. Ethn Heal. 2017;22(1):17–35.

18. Agudelo- AA, Vargas- MY, Vahos- VJ, Ariza- G, Rojas- SWJ, Ronda- GE. A qualitative study of employment , working and health conditions among Venezuelan migrants in Colombia. 2022;(February 2021):1–11.

19. Chávez S, Altman CE. Gambling with life: Masculinity, risk, and danger in the lives of unauthorized migrant roofers. Am J Ind Med. 2017;60(6):537–47.

20. Carlos JK, Wilson K. Migration among temporary foreign workers: Examining health and access to health care among Filipina live-in caregivers. Soc Sci Med [Internet]. 2018;209:117–24. Available from: https://doi.org/10.1016/j.socscimed.2018.05.045

21. Cain P, Daly A, Reid A. How Refugees Experience the Australian Workplace : A Comparative Mixed Methods Study. 2021;

22. Winkelman SB, Chaney EH, Bethel JW. Stress, depression and coping among latino migrant and seasonal farmworkers. Int J Environ Res Public Health. 2013;10(5):1815–30.

23. Ahonen EQ, Porthé V, Vázquez ML, García AM, López-Jacob MJ, Ruiz-Frutos C, et al. A qualitative study about immigrant workers’ perceptions of their working conditions in Spain. J Epidemiol Community Health. 2009;63(11):936–42.

24. Dean JA, Wilson K. ’Education? It is irrelevant to my job now. It makes me very depressed ’: Exploring the health impacts of under/unemployment among highly skilled recent immigrants in Canada. Ethn Heal. 2009;14(2):185–204.

25. Ronda E, Briones-Vozmediano E, Galon T, García AM, Benavides FG, Agudelo-Suárez AA. A qualitative exploration of the impact of the economic recession in Spain on working, living and health conditions: Reflections based on immigrant workers' experiences. Heal Expect. 2016;19(2):416–26.

26. León-pérez G, Non AL. Precarious Work and Parenting Stress among Mexican Immigrant Women in the United States. 2021;83(June):881–97.

27. Nilvarangkul K, Rungreangkulkij S, Wongprom J. Perception of stress in Laotian migrant workers in Thailand. J Immigr Minor Heal. 2010;12(5):678–82.

28. Fleming PJ, Villa-Torres L, Taboada A, Richards C, Barrington C. Marginalisation, discrimination and the health of Latino immigrant day labourers in a central North Carolina community. Heal Soc Care Community. 2017;25(2):527–37.

29. Labonté R, Cobbett E, Orsini M, Spitzer D, Schrecker T, Ruckert A. Globalization and the health of Canadians: "Having a job is the most important thing." Global Health. 2015;11(19):1–16.

30. Martínez AD, Piedramartel A, Agnew J. Going Beyond the Injury: Regulatory Conditions Contributing to Latina/o Immigrants' Occupational Psychosocial Stressors. Front Public Heal. 2015;3:240–8.

31. Robert G, Martínez JM, García AM, Benavides FG, Ronda E. From the boom to the crisis: Changes in employment conditions of immigrants in Spain and their effects on mental health. Eur J Public Health. 2014;24(3):404–9.

32. Panikkar B, Woodin MA, Brugge D, Hyatt R, Gute DM, Community Partners of the Somerville Community Immigrant Worker Project. Characterizing the low wage immigrant workforce: A comparative analysis of the health disparities among selected occupations in Somerville, Massachusetts. Am J Ind Med. 2014;57(5):516–26.
